# Supplementary material for: Shape control in 2D molecular nanosheets by tuning anisotropic intermolecular interactions and assembly kinetics
Source: Nat Commun. 2023 Mar 21;14:1554. doi: 10.1038/s41467-023-37203-7 (PMC10030871; doi:10.1038/s41467-023-37203-7)
Supplement: Supplementary file 1 — Supplementary Information [file 41467_2023_37203_MOESM1_ESM.pdf]

Supplementary Information

Shape control of 2D molecular nanosheets by tuning anisotropic intermolecular interactions and assembly kinetics

Maximilian Dreher [a,†], Pierre Martin Dombrowski [a,†], Matthias Wolfgang Tripp [b], Niels Münster [b], Ulrich Koert [b], Gregor Witte [a,]*

[a] Fachbereich Physik, Philipps-Universität Marburg, Renthof 7, 35032 Marburg, Germany

[b] Fachbereich Chemie, Philipps-Universität Marburg, Hans-Meerwein-Straße 4, 35032 Marburg, Germany

**E-Mail: gregor.witte@physik.uni-marburg.de*

†M.D. and P.M.D. contributed equally to this work.

Contents

Supplementary Note 1: NEXAFS measurements ……………………………………………………… S-2

Supplementary Note 2: Substrate surface quality ………………………………………………… S-3

Supplementary Note 3: Nanosheet shape at different stages of growth ………………… S-4

Supplementary Note 4: Beginning of the desorption process ………………………………. S-5

Supplementary Note 5: Energetically optimised nanosheet shapes ………………………… S-6

Supplementary Note 6: Attachment energy maps ………………………………………………… S-8

Supplementary Note 7: Desorption analysis for different L-F_6PEN nanosheet shapes S-13

Supplementary Note 8: Cohesive energy maps ……………………………………………………… S-14

Supplementary Note 9: Attachment and desorption simulations for L-F_6PER ……………. S-16

Supplementary Note 10: Work function measurements ………………………………………… S-17

Supplementary Note 11: Interaction parameters ………………………………………………… S-18

Supplementary Note 12: Simulated desorption of pentacene ……………………………….. S-21

Supplementary References …………………………………………………………………………… S-22

Supplementary Note 1: NEXAFS measurements

In our theoretical model, we assume that L-F_6PEN and M-F_6PEN molecules adopt a flat-lying orientation in (sub-) monolayers on MoS_2, as indicated by our STM measurements. To independently confirm this adsorption geometry experimentally, we have conducted C1s near-edge x-ray absorption fine structure (NEXAFS) dichroism measurements of a nominal monolayer of L-F_6PEN on MoS_2. As shown in previous work,[1] the low energetic resonances (below 288 eV) of the C1s NEXAFS signature (cf. Suppl. Fig. 1) can be identified as transitions from the C1s core levels into the unoccupied molecular π orbitals (associated with the LUMO, LUMO+1, … levels). Since the transition dipole moments of such low-energy π^* resonances are oriented perpendicular to the molecular ring plane, the molecular orientation can be determined from the intensity of such resonances as a function of the angle of incidence of the incident X-ray radiation.[2] As shown in Suppl. Fig. 1, such dichroism measurements reveal the highest π^* resonance intensity for grazing incidence while they vanish at perpendicular incidence (90°) and thereby confirm a flat lying adsorption geometry of L-F_6PEN on MoS_2.

Although no corresponding measurements were made for M-F_6PEN (sub-) monolayers on MoS_2, our previous work shows that M-F_6PEN also adopts a flat-lying orientation in films deposited on graphite.[3] Because of similar absorption behavior found for fluorinated acenes on graphite and TMDCs,[4,5] a planar adsorption geometry of M-F_6PEN can also anticipated on MoS_2, as evidence by our STM data.

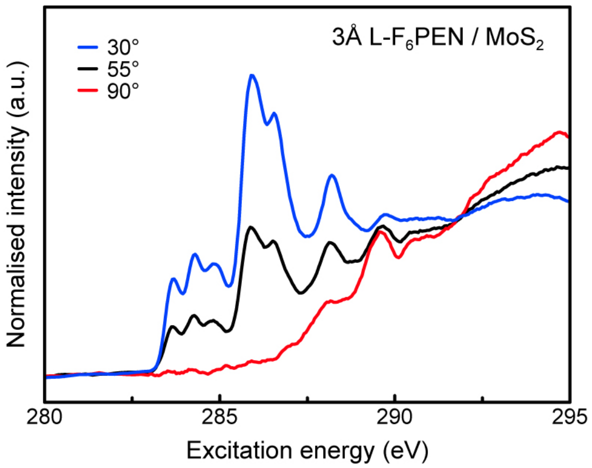

Suppl. Fig. 1 | NEXAFS of L-F_6PEN. C1s NEXAFS spectra of a nominal monolayer (3Å) of L-F_6PEN on MoS_2 recorded at different angles of incidence of the X-ray light with respect to the sample surface. Experiments were conducted in partial electron yield mode, recording the yield of electrons emitted by the sample upon absorption of the x-ray radiation with a retarding field of 150 eV.

Supplementary Note 2: Substrate surface quality

For the present study, freshly exfoliated MoS_2(001) single crystal surfaces were used as substrates for the nanosheets. As shown previously, these yield well-ordered surfaces.[4] While such exfoliated basal planes consist of atomically flat terraces extending over of several tens of microns separated by monoatomic steps, the surfaces also reveal sulphur vacancies, which are characteristic for TMDCs and appear in the STM micrograph as dark depressions due to the reduced electron density (cf. Suppl. Fig. 2a,b).[6] As depicted in Suppl. Fig. 2a, these occur at distances of 5-50 nm. Notably, their presence does not affect the domain size of the molecular adlayer. As shown in Suppl. Fig. 2c for a L-F6PEN monolayer, individual domains can extend over 200 nm and the vacancies of the substrate are not visible in the adlayer.

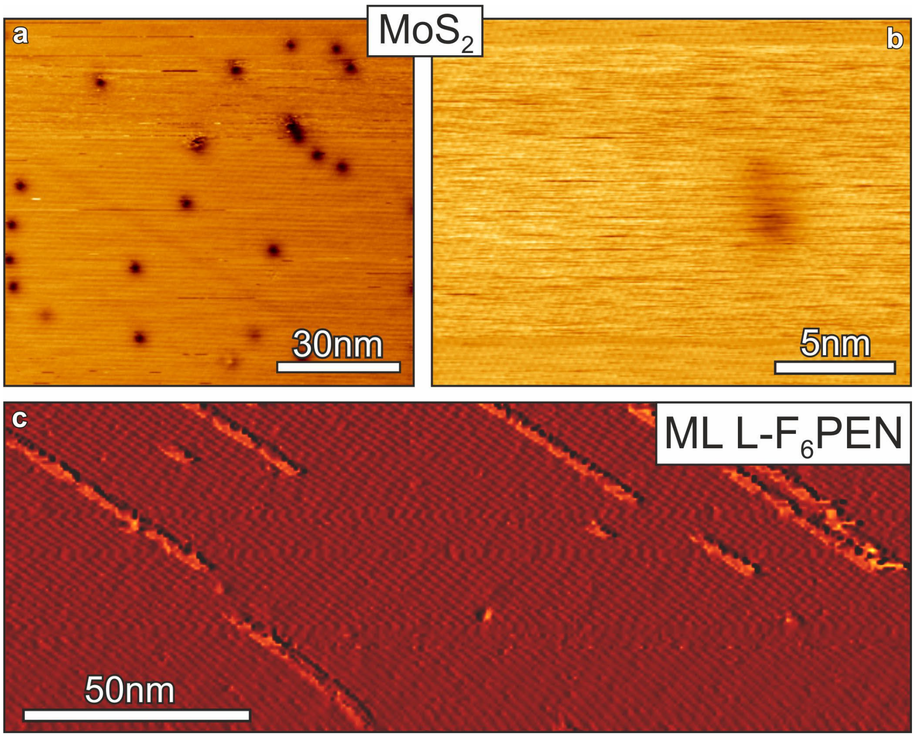

Suppl. Fig. 2 | STM micrographs of MoS_2 and L-F_6PEN on MoS_2. a,b Bare MoS_2(0001) single crystal surface. **c,** Nominal monolayer (3 Å) L-F_6PEN on MoS_2(0001) (U_b = 1.4 V, I_t = 1.4 nA).

Supplementary Note 3: Nanosheet shape at different stages of growth

Since it is highly challenging to image molecular islands on a scale that allows to determine their aspect ratios while achieving molecular resolution, we show only one image for nanosheets formed by adsorption and partial desorption, respectively, in the main paper. In addition, for nanosheets grown by adsorption, individual molecules on the surface that have not attached to a nanosheet are highly mobile even at low temperatures, causing a very unstable tip quality. Suppl. Fig. 3 shows STM images of directly deposited L-F_6PEN films on MoS_2 with different nominal film coverages, in addition to the data shown in Fig. 1d in the main paper.

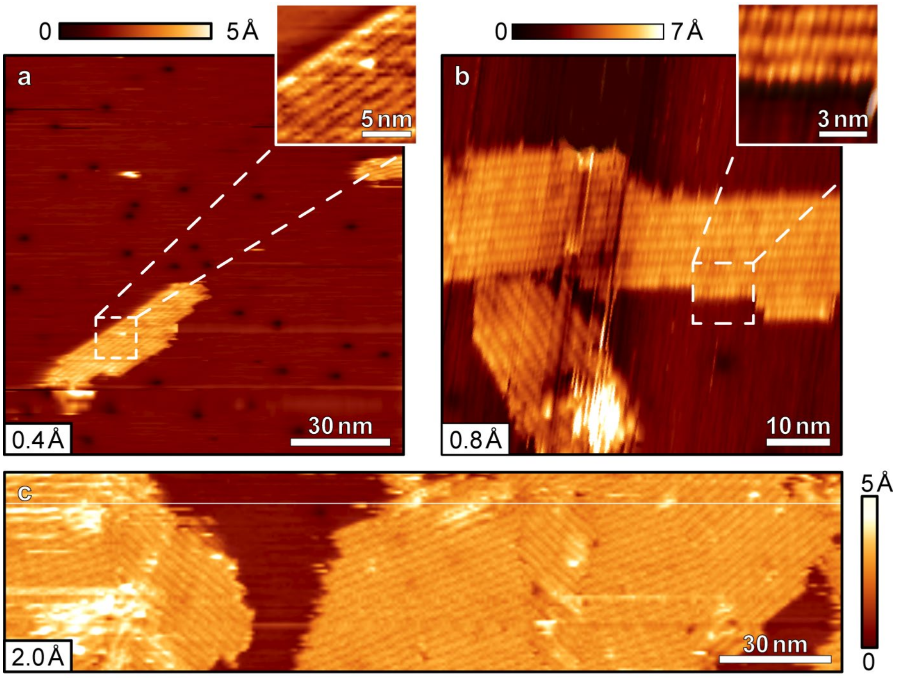

Suppl. Fig. 3 | Island growth of L-F_6PEN. a,b,c STM micrographs (recorded at 110K) of L-F_6PEN film on MoS_2 with a nominal thickness of 0.4Å (U = 1.2 V, I = 430 pA), 0.8Å (U = 1.2 V, I = 40 pA), and 2.0Å (U = 1.2 V, I = 70 pA), respectively. These thicknesses were determined using quartz crystal microbalances.

Suppl. Fig. 3a shows a STM micrograph of a L-F_6PEN film with a nominal thickness of 0.4Å, corresponding to approximately 10% of a nominal monolayer. Consequently, most of the MoS_2 surface is not covered with molecules, which is why MoS_2 surface defects are visible (black spots; cf. Suppl. Fig. 2). Only one L-F_6PEN island and a fraction of a second island at the right edge of the image are present. The island has an area of about 50nm x 15nm, which is comparable to the island shown in Suppl. Fig. 3b (the same image as that in Fig. 1d in the main paper), which shows a film with a nominal thickness of 0.8Å, corresponding to approximately 25% surface coverage. The inset in Suppl. Fig. 3a reveals that the island is extended in direction $\langle\vec{b}\rangle$, in agreement with the island shape observed for the film with half the nominal surface coverage (cf. Suppl. Fig. 3a).
At higher coverages of 2.0Å, approximately 60% of nominal monolayer, we observe coalesced islands, as demonstrated by the STM micrograph in Suppl. Fig. 3c. Therefore, no conclusions can be drawn on preferred directions of nanosheet growth.

Supplementary Note 4: Beginning of the desorption process

Desorption of molecules is most likely to commence with molecules that have the weakest bond to their environment. In the case of our partially fluorinated pentacene derivatives adsorbed on MoS_2, where the dominant attractive forces binding molecules to a film are lateral intermolecular interactions, these are molecules located at nanosheet edges, domain boundaries, or other defects, as such molecules lack attractive nearest-neighbour interactions with other molecules. However, due to the highly ordered growth of the partially fluorinated pentacenes with domains extending over more than 100nm, domain boundaries and other structural defects are relatively sparse.

Suppl. Fig. 4 shows an STM micrograph of a nominal monolayer of L-F_6PEN that was annealed at 400 K for few seconds to activate desorption of some molecules. The image shows no domain boundaries. Yet, molecules have desorbed from within a domain, showing that initial desorption can occur from within the ordered structure if few domain boundaries are present. It is also evident that the desorption of a single molecule from the film creates a vacancy that induces further desorption of molecules in the vicinity, eventually leading to the formation of individual nanosheets. Interestingly, despite the relatively small number of desorbed molecules, it appears that desorption proceeds in direction $\langle\vec{b}\rangle$ once an initial vacancy is formed, which is consistent with our TPD simulations.

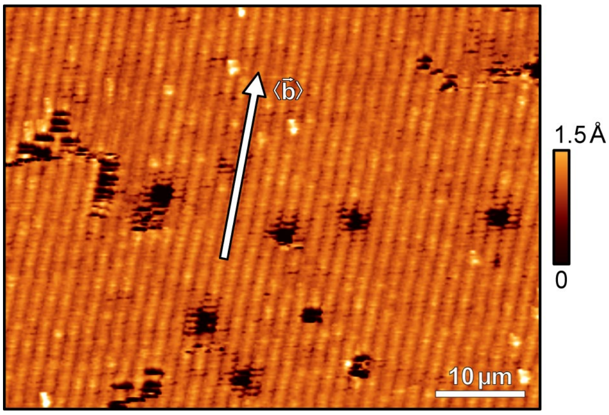

Suppl. Fig. 4 | Beginning desorption. STM image of a nominal monolayer of L-F_6PEN which was annealed to 400K for a couple of seconds (U = 1.2 V, I = 800 pA).

Supplementary Note 5: Energetically optimised nanosheet shapes

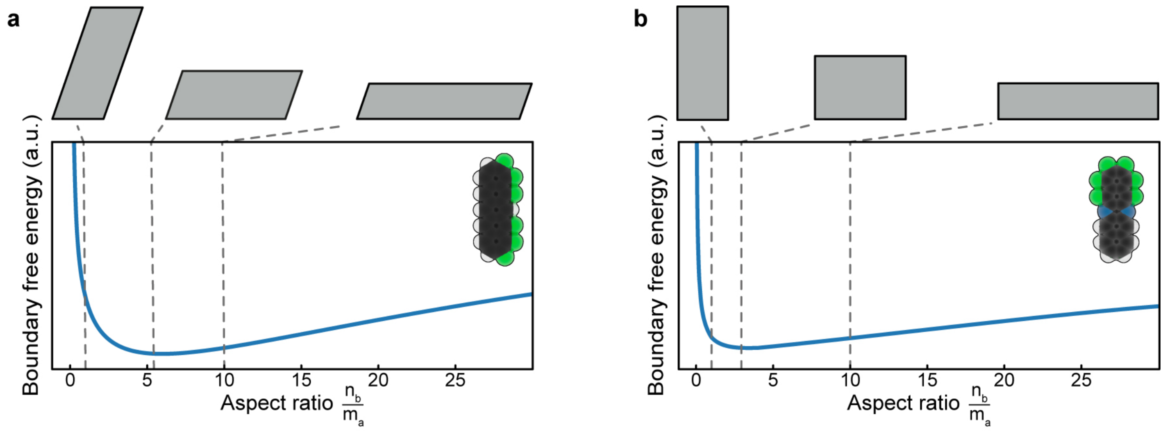

Suppl. Fig. 5 | Boundary free energy of nanosheets. a,b, Boundary free energy of L- and M-F_6PEN nanosheets, respectively, with the same total number of molecules of $m_a \cdot n_b$ as a function of the aspect ratio n_b / m_a and illustrations of nanosheet shapes at exemplary aspect ratios (top).

To determine the energetically optimized nanosheet shape, we have computed the boundary free energy (BFE) of nanosheets as a 2D equivalent of the surface free energy known for 3D crystals. Since we have already calculated cohesive energy maps for nanosheets of L- and M-F_6PEN, we can readily calculate the contribution of a single molecule i at the edge of a nanosheet to its specific BFE, ΔE_i, which is given by the difference between the cohesion energy of a molecule within the nanosheet, E_{bulk}, and the cohesive energy of the considered molecule i at the nanosheet edge, $E_{\mathrm{edge},\,i}$:

$$\Delta E_i = E_{\mathrm{bulk}} - E_{\mathrm{edge},\,i} \;. \qquad \text{(S2)}$$

For a sufficiently large nanosheet, E_{bulk} is the cohesive energy of an arbitrary molecule in the centre of the nanosheet. As the cohesive energy maps in the main paper (cf. Figs. 3a and 4e) show, the cohesive energy is almost constant within the nanosheet and only edge molecules have a lower cohesive energy, so that an approach considering only the edge of the nanosheet separately is valid.

Summation over all edge molecules yields the BFE of a nanosheet $\Delta E_{\mathrm{sheet}}$. For a nanosheet consisting of $m_a \cdot n_b$ molecules / unit cells, one finds

$$\Delta E_{\mathrm{sheet}} = 2(m_{\mathrm{a}}\, \Delta E_{\mathrm{LE}} + n_{\mathrm{b}}\, \Delta E_{\mathrm{SE}})\,, \qquad \text{(S3)}$$

where ΔE_{LE} and ΔE_{SE} are the specific BFEs for a single molecule at an *LE* or *SE*, respectively, according to eqn. S2. For L-F_6PEN, we find ΔE_{LE} = 97 kJ mol^{-1} = 1.01 eV and ΔE_{SE} = 18 kJ mol^{-1} = 0.187 eV. For M-F_6PEN, we find ΔE_{LE} = 29 kJ mol^{-1} = 0.301 eV and ΔE_{SE} = 9 kJ mol^{-1} = 0.093 eV.

To understand what type of island shape is energetically preferred, the BFE, $\Delta E_{\mathrm{sheet}}$, can be calculated as a function of the nanosheet index aspect ratio n_b/m_a. For a fixed number of 10^8 molecules, all integer pairs of m_a and n_b fulfilling $m_a \cdot n_b = 10^8$ were identified and used to calculate $\Delta E_{\mathrm{sheet}}$ for the corresponding aspect ratios. Suppl. Figs. 5a and b show the corresponding plots of $\Delta E_{\mathrm{sheet}}$ for L- and M-F_6PEN, respectively, that were interpolated to obtain a continuous curve. Interestingly, both curves are of a similar shape. They exhibit a shallow absolute minimum, corresponding to the energetically optimized sheet shape, at around $n_b/m_a \approx 5.5$ in the case of L-F_6PEN and $n_b/m_a \approx 3$ in the case of M-F_6PEN. Towards smaller aspect ratios, i.e., towards $\langle\vec{a}\rangle$-extension, both curves feature a steep flank that appears to diverge in the limit of an aspect ratio of zero. Towards larger aspect ratios and thus more pronounced $\langle\vec{b}\rangle$-extension, the curves exhibit a much less steep increase of the BFE.

The exact minimum of the BFE can be determined analytically from eqn. S3. For a fixed number of molecules of N = $m_a \cdot n_b$, we can substitute m_a = N / n_b and then find the zero of the derivative $\partial(\Delta E_{\text{sheet}})/\partial n_b$. Thus, one finds that the optimum index aspect ratio at which the BFE of the sheet is minimum is given by the ratio of the specific BFEs:

$$\frac{n_{\text{b}}^{\text{opt}}}{m_{\text{a}}^{\text{opt}}} = \frac{\Delta E_{\text{LE}}}{\Delta E_{\text{SE}}} \; . \qquad \text{(S4)}$$

For L-F_6PEN, eqn. S4 yields an optimum index aspect ratio of 5.38, in good agreement with Suppl. Fig. 5a. This index aspect ratio corresponds to a geometrical aspect ratio of 1:2.35 ($\langle\vec{a}\rangle$:$\langle\vec{b}\rangle$) and thus clearly to a preference for $\langle\vec{b}\rangle$-extension. For M-F_6PEN, eqn. S4 yields an index aspect ratio of 3.22, corresponding to a geometrical aspect ratio of 1:1.44 ($\langle\vec{a}\rangle$:$\langle\vec{b}\rangle$), which is only a slightly $\langle\vec{b}\rangle$-extended sheet.

Regarding our experimental results, the above calculations show clearly that the experimentally observed sheet growth is kinetically controlled, as neither adsorption nor desorption yield nanosheets with the energetically optimised aspect ratio. Due to strong intermolecular interactions, adsorption as well as desorption processes are governed by local nearest-neighbour interactions rather than mesoscopic optimization of the BFE and thus the total energy of the sheet.

Supplementary Note 6: Attachment energy maps

In the main paper, attachment energy maps (AEMs) are only shown for equally oriented molecules. In addition to those AEMs, we have also calculated AEMs for relative angles of 45°, 90°, 135°, and 180° that provide deeper insight into intermolecular interactions and preferred structural configurations, in particular upon direct comparison of L-F_6PEN, M-F_6PEN, and L-F_6PER.

Suppl. Fig. 6 shows AEMs for pair interactions of L-F_6PEN (Suppl. Fig. 6a-e), M-F_6PEN (Suppl. Fig. 6f-j), and L-F_6PER (Suppl. Fig. 6k-o). For L-F_6PEN, it is obvious that a relative angle of 0° (Suppl. Fig. 6a), i.e., equally oriented molecules, is the energetically preferred relative orientation, since the strongest attachment energy (negative potential energy) is found here at a relative displacement of approximately Δy = 0. The regime of attractive intermolecular interactions (blue) extends only to LE attachment sites, whereas around the SE, interactions are repulsive (red). Therefore, LE attachment is clearly favoured even when only two molecules are considered. At a relative angle of 180° (Suppl. Fig. 6e), the AEM is basically inverted, showing strong repulsion at the LE and slight attraction at the SE. However, this attraction is weaker than that at the LE for a relative angle of 0°, and the attractive regions are much smaller than the repulsive ones, so that molecules are most likely to rotate rather than attach at the SE. This can also be seen in Suppl. Figs. 6b and c, showing that the size of the attractive regions increase as the relative angle comes closer to 0°. Thus, L-F_6PEN molecules are driven towards a relative angle of 0° and then towards attachment along the LE.

For M-F_6PEN (Suppl. Fig. 6f-j), intermolecular interactions are overall weaker. Strongest attraction occurs at a relative angle of 180° (Suppl. Fig. 6j), in line with the experimental observation that LE-neighbouring M-F_6PEN molecules alternate in their orientation by 180° (cf. Fig. 4a in the main paper). There is also considerable attraction at the SE for a relative angle of 0° (Suppl. Fig. 6f) at a relative displacement of Δx = 0, supporting the proposed orientation of molecules within a nanosheet depicted in Fig. 4d in the main paper.

For L-F_6PER (Suppl. Fig. 6k-o), the AEMs look quite similar to those of L-F_6PEN. The single-molecule AEMs indicate that a uniform molecular packing motif with a relative angle of 0° between neighbouring molecules is the most stable configuration. However, an alternating orientation with a relative angle of 180° still shows areas of significant intermolecular attraction, with a difference to a relative angle of 0° that is much smaller than those observed for the pentacene derivatives. This indicates that both structures could be stable.

Suppl. Fig. 7a shows an AEM for a nanosheet of 3x4 L-F_6PEN, probed by an L-F_6PEN molecule with the same orientation as the molecules that are part of the nanosheet. As in the corresponding AEM of a single molecule (Suppl. Fig. 6a), strongest attraction is observed along the LEs. However, the AEM of the nanosheet also exhibits attractive regions along the SEs, located at virtual lattice points of the nanosheet. Suppl. Fig. 7b shows a magnification of the corresponding area, where these binding regions are more clearly visible and highlighted by a black outline. Though their overall attraction is significantly weaker in comparison to the attractive interactions along the LEs, these regions do allow molecules to attach at the SEs if they can overcome the surrounding potential barrier. Thus, island growth can also proceed in direction <b>, though the energy gain for the system is significantly smaller. Due to the repulsive barrier around the SEs, attachment of molecules is more likely to occur at the LEs with stronger and long-ranged attractive forces, in line with our kinetic Monte Carlo simulations.

AEMs of island sheets probed by L-F_6PEN molecules with varying orientation are shown in Suppl. Fig. 8 for L-F_6PEN and M-F_6PEN, and in Suppl. Fig. 9 for L-F_6PER. As in the case of the single-molecule AEMs (Suppl. Fig. 6a-e), strongest attraction for L-F_6PEN (Suppl. Fig. 8a-e) is again observed at LE sites for equal orientation of the attaching molecule and the molecules within the nanosheet, i.e., at a relative angle of 0° (Suppl. Fig. 8a). A relative angle of 180° leads essentially to an inversion of the AEM (Suppl. Fig. 8e). Though the AEM is attractive along the SE, attachment in an inverted orientation relative to that of molecules within the nanosheet prohibits continuation of the lattice upon further growth and is therefore energetically disadvantageous. Instead, the large repulsive regions are likely to induce rotation of the approaching molecule until the favoured relative angle of 0° is achieved.

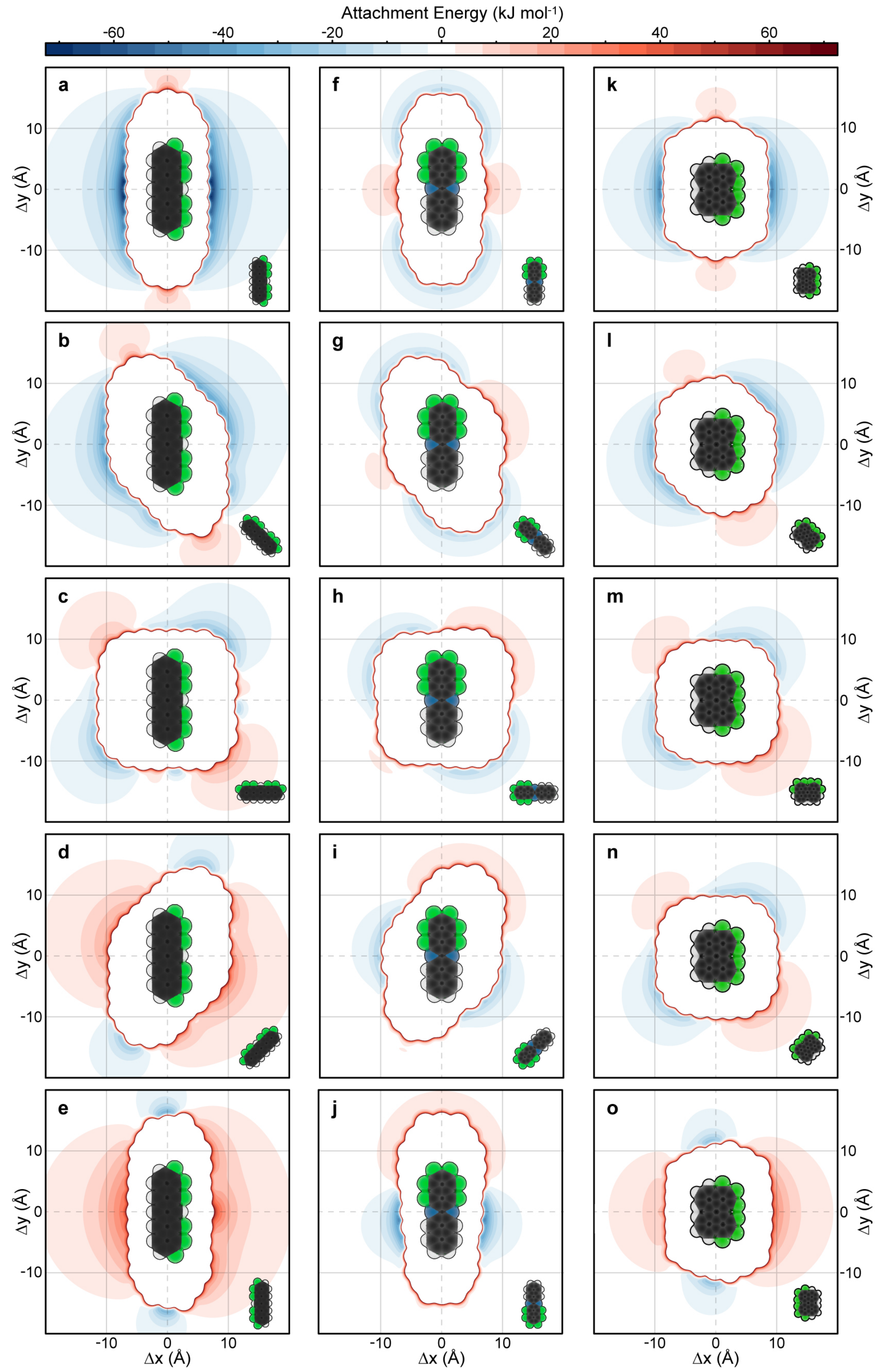

Suppl. Fig. 6 | AEMs for single molecules. a-e, Attachment energy maps (AEMs) for two L-F_6PEN molecules at relative angles of 0°, 45°, 90°, 135°, and 180°, respectively, as indicated by the small molecule in the bottom right corner of each map. **f-j,** Corresponding AEMs for M-F_6PEN. **k-o,** Corresponding AEMs for L-F_6PER.

In comparison to the AEMs of L-F_6PEN, the AEMs of M-F_6PEN nanosheets (Suppl. Fig. 8f-j) show some striking differences. Firstly, we note that the energy scale is reduced to less than half the range of that of L-F_6PEN. Despite the reduced energy scale and thus higher energy-resolution and contrast, the interaction range of the M-F_6PEN nanosheet is much smaller, extending over only few Å. An explanation for the smaller interaction range can be found in the orientation of molecules within the nanosheet: L-F_6PEN molecules are all equally oriented, which amplifies the single-molecule interaction potentials. By contrast, the alternating orientation of M-F_6PEN molecules within the nanosheet leads to a reduction of the interaction potential, both in magnitude and in range.
Another important difference is that for the relative angles of 0° or 180° for attaching molecules (Suppl. Fig. 8f and j, respectively), there are alternating regions of attraction and repulsion along the LE, which further reduces the interaction range. By contrast, the SE features larger regions of attraction that extend further away from the island. Therefore, even though the maximum SE binding energy is smaller than the LE binding energy, molecules are more likely to attach at SE sites, as we see in the dynamic attachment simulations (cf. Fig. 3e in the main paper).
For L-F_6PER, AEMs were computed for a uniform molecular packing motif like that of L-F_6PEN (cf. Suppl. Fig. 9a-e) and for an alternating packing motif similar to that of M-F_6PEN (cf. Suppl. Fig. 9f-j). Obviously, the AEM strongly depends on the packing motif. However, on a qualitative level, the AEMs of L-F_6PER closely resemble those of the pentacene derivatives for the respective packing motifs.

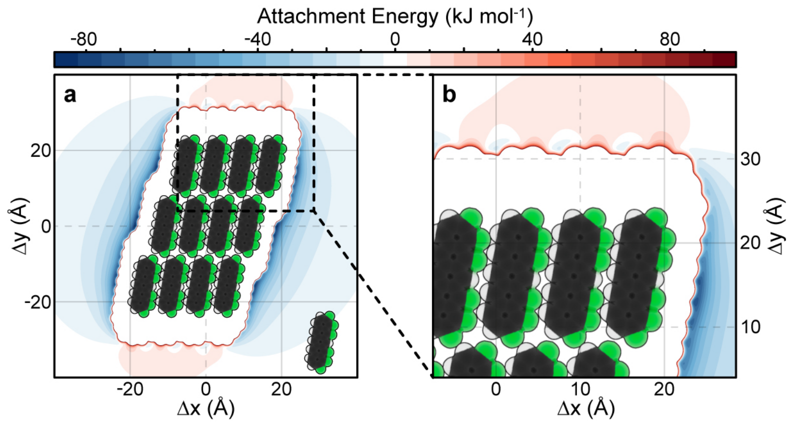

Fig. S7 | AEM of an L-F_6PEN nanosheet. a, Attachment energy map (AEM) of an L-F_6PEN nanosheet, probed by an equally oriented L-F_6PEN molecule as indicated by the molecule in the bottom right corner. **b,** Magnification of the AEM, showing the cusps of attractively interacting areas (blue) that are surrounded by a repulsive barrier (red) along the SE.

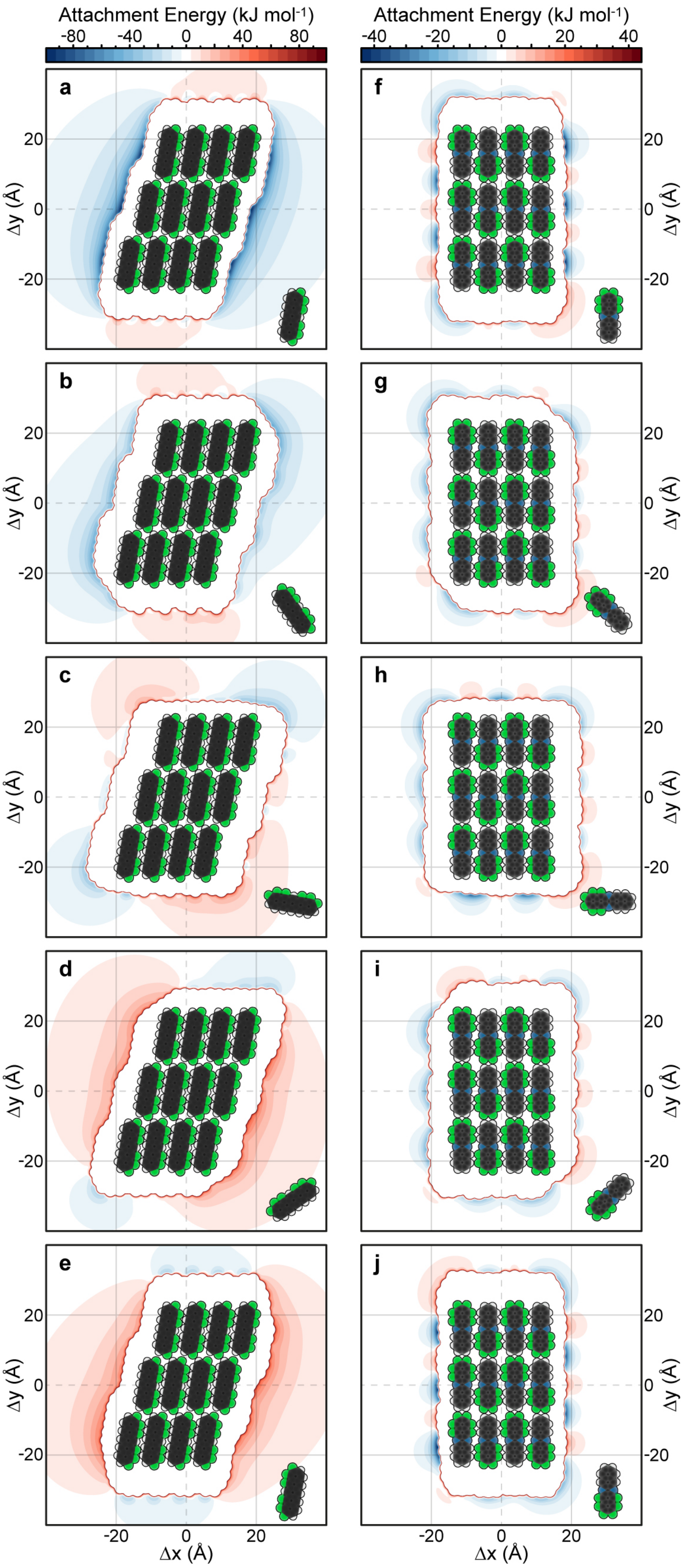

Suppl. Fig. 8 | AEMs for nanosheets of L- and M-F_6PEN. a-e, Attachment energy maps (AEMs) for L-F_6PEN nanosheets, probed single molecules at relative angles of 0°, 45°, 90°, 135°, and 180°, respectively, as indicated in by the small molecules in the bottom right corner of each map. **f-j,** Corresponding AEMs for M-F_6PEN.

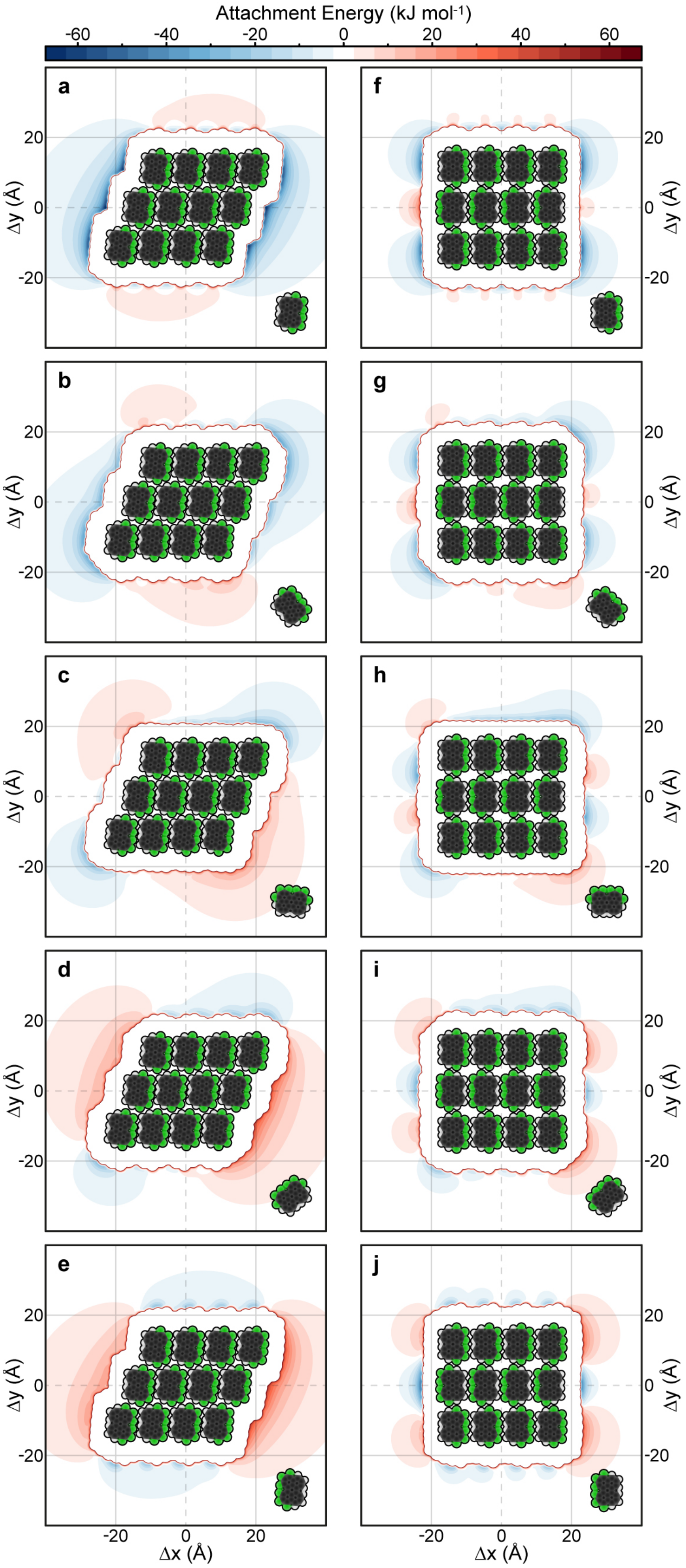

Suppl. Fig. 9 | AEMs for L-F_6PER nanosheets. a-e, Attachment energy maps (AEMs) for L-F_6PER nanosheets with a uniform packing motif, probed single molecules at relative angles of 0°, 45°, 90°, 135°, and 180°, respectively, as indicated by the small molecule in the bottom right corner of each map. **f-j,** Corresponding AEMs for L-F_6PER nanosheets with an alternating packing motif.

Supplementary Note 7: Desorption analysis for different L-F_6PEN nanosheet shapes

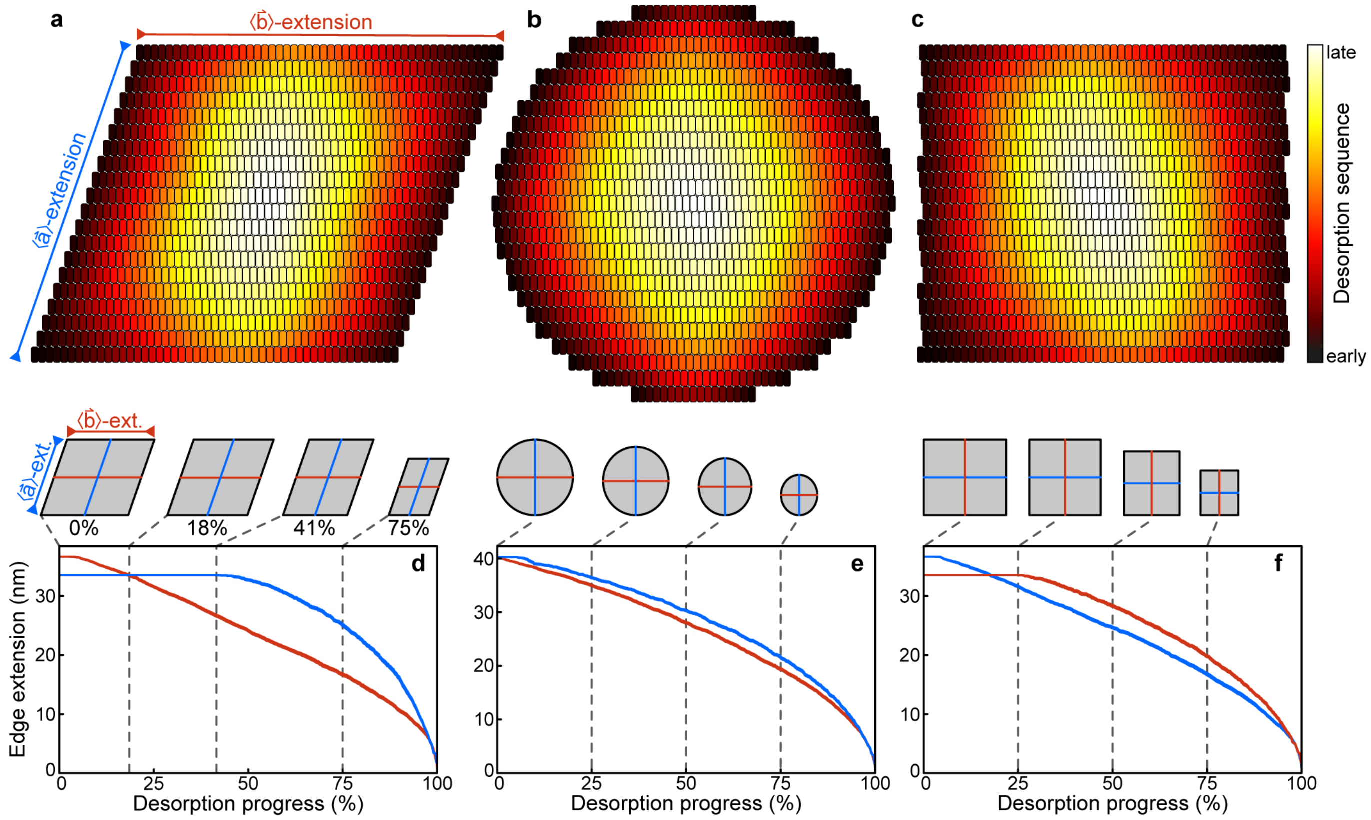

Suppl. Fig. 10 | Desorption simulation for different L-F_6PEN nanosheets. a,b,c Maps of the desorption sequence of L-F_6PEN for initially oblique, circular, and rectangular nanosheets, respectively. **d,e,f** Corresponding plots of the $\langle\vec{a}\rangle$- (blue) and $\langle\vec{b}\rangle$- (red) extensions during the complete desorption process. All maps were averaged over 50 simulated temperature-programmed desorption experiments.

To prove that the preference for $\langle\vec{a}\rangle$-extension upon partial desorption of nanosheets observed in our simulations does not depend on the chosen initial shape of the nanosheet, we have conducted additional simulations for other initial nanosheet shapes. Suppl. Fig. 10a shows the desorption sequence map for an oblique nanosheets that is also shown and discussed in the main paper. Suppl. Figs. 10b and c show the corresponding maps for a circular and a rectangular nanosheet, respectively. From these maps, we can already see that for all initial shapes, the inner, yellowish shapes, corresponding to a desorption progress of > 60%, exhibit a larger $\langle\vec{a}\rangle$- than $\langle\vec{b}\rangle$-extension. Although the initial nanosheet shape does influence the later shape, the observed preference for $\langle\vec{a}\rangle$-extension is reproduced for all tested initial shapes.

This is corroborated by plots of the maximum $\langle\vec{a}\rangle$- (blue) and $\langle\vec{b}\rangle$- (red) extension of the island during the desorption simulation shown in Suppl. Fig. 10d-f. In all three cases, the nanosheets quickly adopt a larger $\langle\vec{a}\rangle$- than $\langle\vec{b}\rangle$-extension even though the initial $\langle\vec{a}\rangle$-extension is slightly lower than or equal to the initial $\langle\vec{b}\rangle$-extension. For the initially oblique (Suppl. Fig. 10d) and rectangular (Suppl. Fig. 10f) nanosheets, this effect is most clear due to the $\langle\vec{a}\rangle$-extension remaining constant during initial desorption while the $\langle\vec{b}\rangle$-extension continuously decreases. For the initially circular nanosheets, this effect is not clear due to initially equal $\langle\vec{b}\rangle$- and $\langle\vec{a}\rangle$-extensions. Nonetheless, as desorption commences, the $\langle\vec{a}\rangle$-extension is always larger than the $\langle\vec{b}\rangle$-extension. Thus, we can conclude that the initial nanosheet shape does not significantly influence the outcome of our simulations that $\langle\vec{a}\rangle$-extended are preferred upon partial desorption of nanosheets.

Supplementary Note 8: Cohesive energy maps

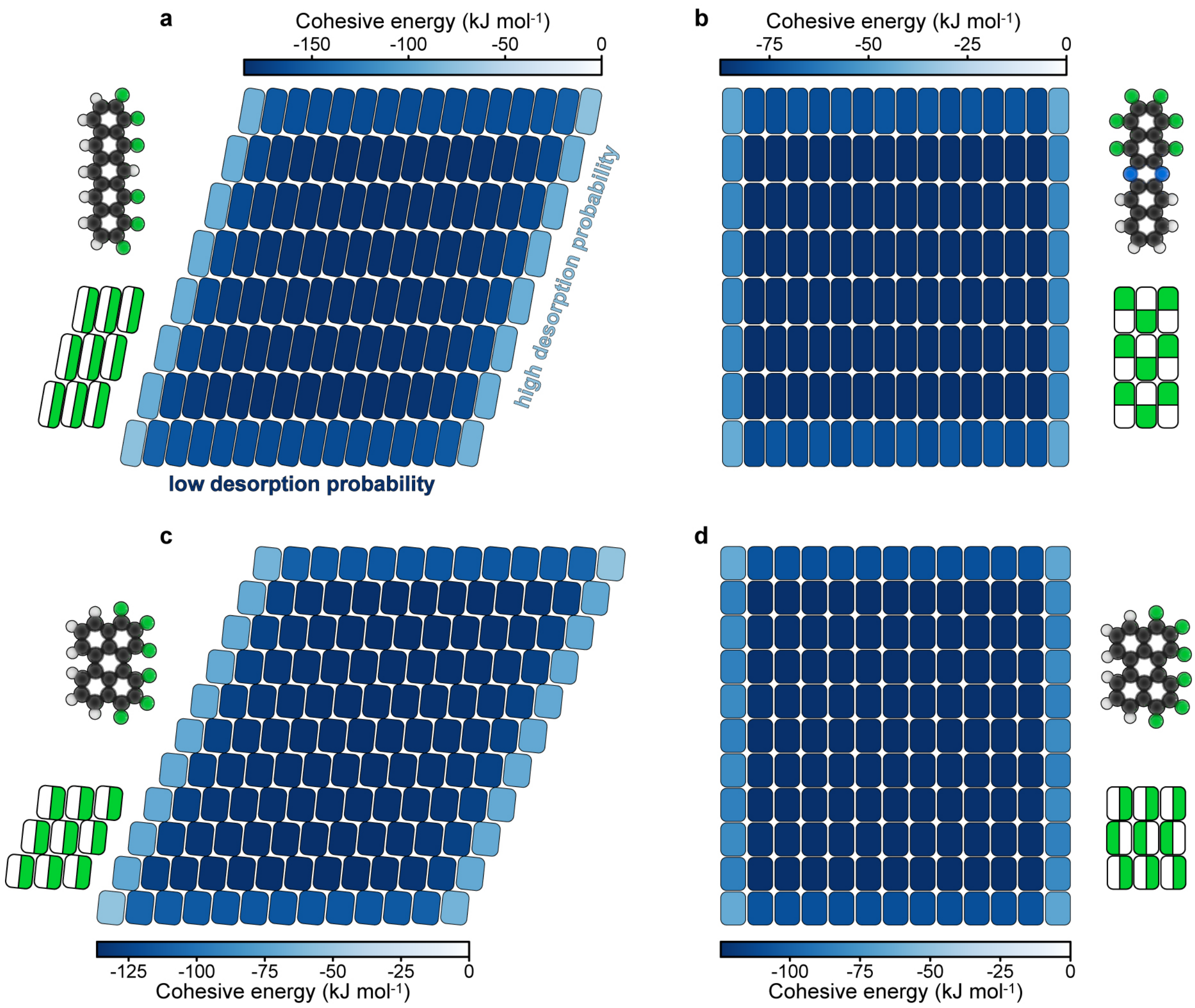

Suppl. Fig. 11 | Cohesive energy maps. a, Cohesive energy map (CEM) for an 8x16 nanosheet of L-F_6PEN. The colour of a molecule denotes its binding energy due to interactions with all other molecules. Darker colours correspond to stronger binding. **b,** Corresponding CEM for M-F_6PEN. **c,d,** CEMs for L-F_6PER in a uniform packing motif (see bottom left illustration) and in an alternating packing motif (see bottom right illustration), respectively, for an 11x13 nanosheet.

To fully understand the kinetics of desorption of molecules from a nanosheet, one must consider the cohesive energy of molecules in a nanosheet, that is the energy that binds a molecule to the sheet. Suppl. Fig. 11 shows cohesive energy maps (CEMs) for all molecules and structures analysed in this study, for one exemplary nanosheet shape. These maps were generated by computation of the potential energies of all molecules in the nanosheet due to interactions with all other molecules.

For L-F_6PEN (cf. Suppl. Fig. 11a), the CEM clearly shows that molecules located at the LEs of the nanosheet have a significantly smaller cohesive energy than all other molecules, approximately 40% of the cohesive energy of a molecule from the centre of the nanosheet. By contrast, the cohesive energy of molecules located at the SEs is significantly larger and much closer to that of a centre molecule with approximately 90% of the cohesive energy of a centre molecule. This is due to the anisotropic intermolecular interactions that are much stronger in direction $\langle\vec{b}\rangle$ than in direction $\langle\vec{a}\rangle$. Molecules located at the *LEs* therefore lack one of their two strongest attracting nearest neighbours, whereas those located at the SEs lack only a weakly attracting neighbour (cf. Suppl. Figs. 6a and 7a). Therefore, the likelihood of desorption is highest for molecules located at these edges, in agreement with the results of the TPD simulations.

For M-F_6PEN (cf. Suppl. Fig. 11b), the relative and absolute difference in cohesive energy of molecules located at the SEs *vs.* LEs is much smaller (90% *vs.* 70%). Hence, the likelihood of desorption from those edges is much closer, explaining the absence of a clear preference for desorption from specific edges. The by far smallest cohesive energy is found at the nanosheet edges with approximately 50% of the cohesive energy of a centre molecule, rendering desorption from edges or, more generally, sites with only two nearest-neighbours, most likely.
For L-F_6PER, the cohesive energy at the nanosheet edges strongly depends on the molecular packing motif. For a uniform packing motif (cf. Suppl. Fig. 11c), the CEM is qualitatively similar to that of L-F_6PEN. For an alternating packing motif (cf. Suppl. Fig. 11d), the CEM is similar to that of M-F_6PEN. Therefore, the discussions of the CEMs of the respective pentacene derivatives can be directly applied to the L-F_6PER structures.

Supplementary Note 9: Attachment and desorption simulations for L-F_6PER

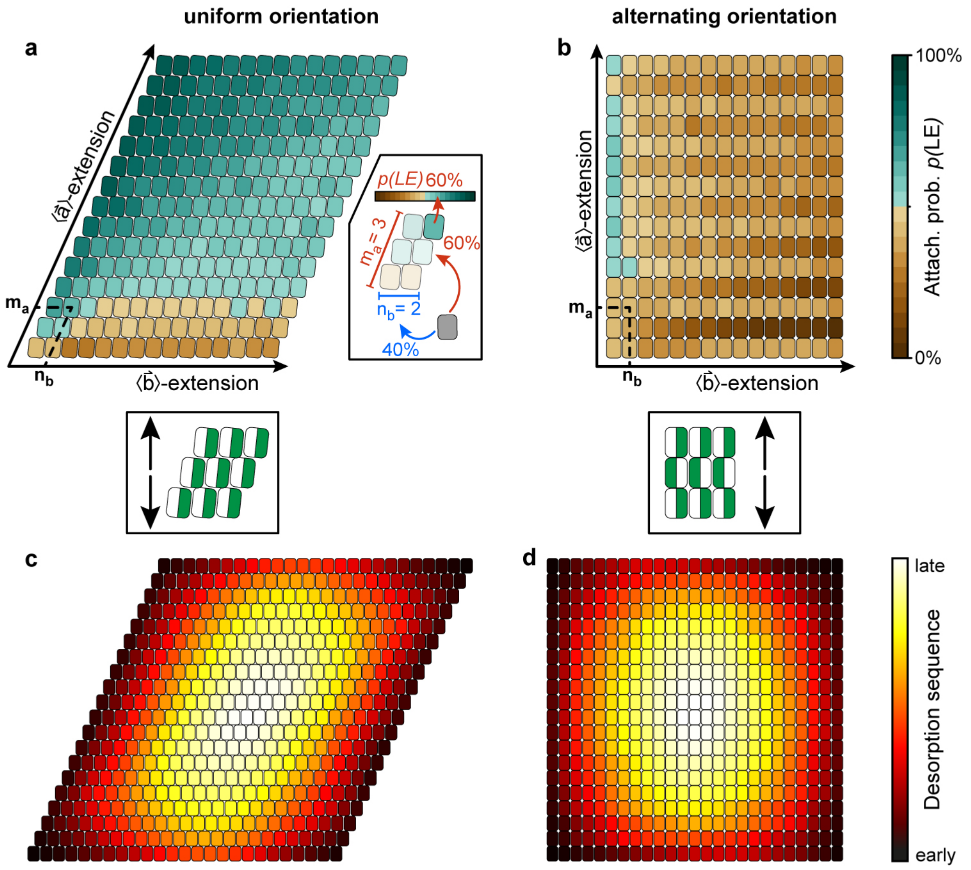

Suppl. Fig. 12 | Attachment and desorption simulations for L-F_6PER. a,b Probability *p*(LE*)* for attachment of an L-F_6PER admolecule to the long molecular side edges (LEs) for a given island shape for a uniform and an alternating molecular packing motif, respectively. Note that *p*(LE*)* = 1 – *p(*SE*)*. **c,d** Maps of the desorption sequence of L-F_6PER molecules from nanosheets with a uniform and alternating molecular packing motif, respectively. Desorption sequences were averaged over 30 simulated temperature-programmed desorption experiments.

Suppl. Fig. 12 shows the results of Monte Carlo ad- and desorption simulations for L-F_6PER with a uniform and alternating packing motif. For the uniform packing motif (cf. Suppl. Figs. 12a and c), the results closely resemble those obtained for L-F_6PEN (cf. Fig. 2 in the main paper): The single-molecule adsorption probability at LEs (cf. Suppl. Fig. 12a) is mostly larger than 50% with the exception highly $\langle\vec{b}\rangle$-extended nanosheets, leading to a preference for the formation of $\langle\vec{b}\rangle$-extended nanosheets upon growth. By contrast, $\langle\vec{a}\rangle$-extension is favoured upon partial desorption of a nanosheet (cf. Suppl. Fig. 12c).
For the alternating packing motif (Suppl. Figs. 12b and d), the results are similar to those found for M-F_6PEN (cf. Fig. 3 in the main paper): Upon attachment of a single molecule, $\langle\vec{a}\rangle$-extension is favoured, whereas no preferential extension is found upon partial desorption of a nanosheet. The finding of a preference for $\langle\vec{a}\rangle$-extension upon adsorption is somewhat surprising, since the direction of alternation of molecular is different to that for M-F_6PEN. For the latter, molecular orientation alternates in direction $\langle\vec{b}\rangle$, whereas the orientation of neighbouring L-F_6PER molecules alternates in direction $\langle\vec{a}\rangle$. Due to this difference, one would expect that L-F_6PER should show preferential nanosheet growth in direction $\langle\vec{b}\rangle$. However, it appears that differences in the AEMs due to the more compact shape of L-F_6PER lead to somewhat different growth kinetics.

Supplementary Note 10: Work function measurements

In our MD simulations of nanosheet formation and desorption, we consider only mutual vdW forces between the molecules without specific substrate interactions, which requires justification. To check whether the adsorption of molecules on the MoS_2 substrate is accompanied by charge transfer due to the molecule-substrate interaction, Kelvin probe work function measurements were performed for the bare MoS_2 surface and the monolayers of L-F_6PEN and M-F_6PEN on MoS_2, which are listed in Suppl. Tab. 1. Since in contrast to metal surfaces, there is no push-back effect [7] upon molecular adsorption on MoS_2, the absence of any work function change confirms that there is no additional charge transfer between MoS_2 and the adsorbed molecular films. This confirms our assumption of a weak molecule-substrate interaction, which is clearly exceeded by the lateral interaction between the molecules.

Suppl. Tab. 1 | Work functions. Kelvin probe measurements of the work function ϕ of L-F_6PEN and M-F_6PEN monolayers on MoS_2.

	ϕ (eV)
MoS_2	4.60 ± 0.05
MoS_2 + 3Å L-F_6PEN	4.61 ± 0.05
MoS_2 + 3Å M-F_6PEN	4.58 ± 0.05

Supplementary Note 11: Interaction parameters

As described in the *Methods* section of the main paper, all intermolecular interactions were treated in an atomistic model. Therefore, optimised molecular structures were calculated by means of density functional theory (DFT) using GAMESS [8] (version 2020.R2) with a B3LYP functional and aug-cc-pVTZ basis set. Based on atomic basis sets from these DFT calculations, natural bond orbital (NBO) analyses were performed using NBO 7.0 [9] to assign each atom of a molecule an effective charge for Coulomb interactions. The thus determined atomic coordinates and charges of L-F_6PEN, M-F_6PEN, L-F_6PER, and pentacene (PEN) are listed in Suppl. Tabs. 2, 3, 4, and 5, respectively, for symmetry-inequivalent atoms, together with labelled illustrations of the corresponding molecules. The corresponding labels for the individual atoms of each molecule are depicted in Suppl. Fig. 13.

Suppl. Tab. 2 | Atomic coordinates and NBO charges for L-F_6PEN.

Atom	Element	x (Å)	y (Å)	q (e)
**1**	C	-1.4027	0.0000	0.2521
**2**	C	-0.7261	1.2218	0.0069
**3**	C	-1.3985	2.4592	-0.1571
**4**	C	-0.7183	3.6626	0.0537
**5**	C	-1.4144	4.9135	-0.2123
**6**	C	-0.7455	6.0940	-0.2420
**7**	C	0.6725	6.0974	0.3337
**8**	C	1.3918	4.9547	0.3587
**9**	C	0.7378	3.6728	-0.2105
**10**	C	1.3908	2.4536	0.4040
**11**	C	0.7228	1.2124	-0.0176
**12**	C	1.4144	0.0000	-0.1574
**13**	H	-2.4857	0.0000	0.2020
**14**	H	-2.4810	2.4670	0.1954
**15**	H	-2.4957	4.8999	0.2103
**16**	H	-1.2573	7.0461	0.2268
**17**	F	1.3080	7.2807	-0.3201
**18**	F	2.7289	5.0113	-0.3103
**19**	F	2.7344	2.4142	-0.3253
**20**	H	2.4929	0.0000	0.2189

Suppl. Tab. 3 | Atomic coordinates and NBO charges for M-F_6PEN.

Atom	Element	x (Å)	y (Å)	q (e)
**1**	N	-1.4327	0.0000	-0.3900
**2**	C	-0.7267	1.1373	0.0265
**3**	C	-1.3882	2.3794	0.5132
**4**	C	-0.7258	3.5914	-0.2033
**5**	C	-1.3902	4.8581	0.3545
**6**	C	-0.7027	6.0160	0.3005
**7**	C	0.7345	-1.1441	0.1158
**8**	C	1.4235	-2.3712	-0.1115
**9**	C	0.7430	-3.5704	-0.0559
**10**	C	1.4341	-4.8276	-0.1262
**11**	C	0.7550	-5.9963	-0.1711
**12**	F	-2.7288	2.3543	-0.3162
**13**	F	-2.7308	4.9042	-0.3042
**14**	F	-1.3171	7.2014	-0.2956
**15**	H	2.3727	-2.3739	0.1903
**16**	H	2.3834	-4.8391	0.1784
**17**	H	1.2308	-6.8171	0.1814

Suppl. Tab. 4 | Atomic coordinates and NBO charges for L-F_6PER.

Atom	Element	x (Å)	y (Å)	q (e)
**1**	C	-2.4301	2.8145	-0.1956
**2**	C	-1.2608	3.5527	-0.1852
**3**	C	-0.0287	2.8686	-0.1502
**4**	C	1.1660	3.6103	0.3684
**5**	C	2.3648	2.9761	0.2953
**6**	C	2.4290	1.5697	0.3451
**7**	C	1.3093	0.7424	-0.0931
**8**	C	0.0390	1.4362	0.0982
**9**	C	-1.1995	0.7320	0.0133
**10**	C	-2.3903	1.4471	-0.2085
**11**	H	-3.3324	0.9237	0.2020
**12**	H	-3.3839	3.3510	0.2105
**13**	H	-1.2602	4.6322	0.2223
**14**	F	1.1208	4.9487	-0.3147
**15**	F	3.5076	3.6660	-0.3029
**16**	F	3.6917	1.1411	-0.3048

Suppl. Tab. 5 | Atomic coordinates and NBO charges for PEN.

Atom	Element	x (Å)	y (Å)	q (e)
**1**	C	-1.4034	0.0000	-0.1871
**2**	C	-0.7264	1.2219	0.0021
**3**	C	-1.4033	2.4591	-0.1130
**4**	C	-0.7257	3.6649	-0.0945
**5**	C	-1.4057	4.9251	-0.1181
**6**	C	-0.7148	6.0955	-0.2325
**7**	H	-2.4875	0.0000	0.1175
**8**	H	-2.4871	2.4598	0.1852
**9**	H	-2.4887	4.9259	0.1911
**10**	H	-1.2432	7.0396	0.2046

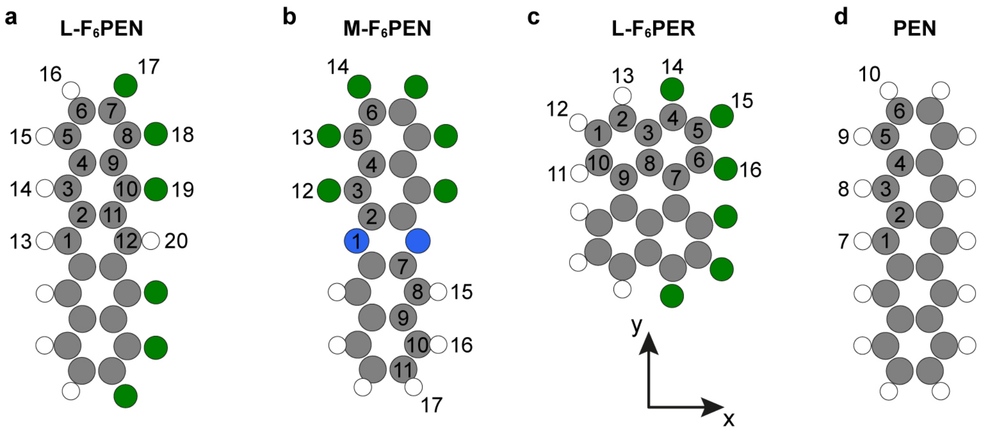

Suppl. Fig. 13 | Atomic labels for all molecules. a,b,c,d Labels for all atoms of L-F_6PEN, M-F_6PEN, L-F_6PER, and PEN. Symmetry-equivalent atoms are not labelled.

Pauli repulsion and dispersion interactions were modelled by a Buckingham potential of the form

$$V_{\mathrm{B}} = a_{ij} e^{-b_{ij} r_{ij}} - \frac{c_{ij}}{r_{ij}^6} \qquad \text{(S1)}$$

for the interaction of two atoms labelled i and j. Here, a_{ij}, b_{ij}, and c_{ij} are element-specific interaction parameters, and r_{ij} is the distance of the interacting atoms. For symmetric interactions between atoms of the same element, interaction parameters for C, H, and N were taken from Ref. 10. Parameters for F interactions were taken from Ref. 11. The symmetric interaction parameters are listed in Suppl. Tab. 6. For asymmetric interactions between different elements, interaction parameters were approximated by the geometric mean of the corresponding symmetric parameters: $a_{ij} = \sqrt{a_{ii} a_{jj}}$, b_{ij} and c_{ij} accordingly.

Suppl. Tab. 6 | Pauli and dispersion interaction parameters. Interaction parameters for symmetric Pauli and dispersion interactions according to eqn. S1. Values for H-H, C-C, and N-N interactions were taken from Ref. 10, those for F-F interactions from Ref. 11.

Element	a (kJ mol^{-1})	b (Å^{-1})	c (Å^6 kJ mol^{-1})
**H**	41680	4.52	181
**C**	3801520	4.59	1515
**N**	868370	4.59	1270
**F**	251900	4.60	494

Supplementary Note 12: Simulated desorption of pentacene

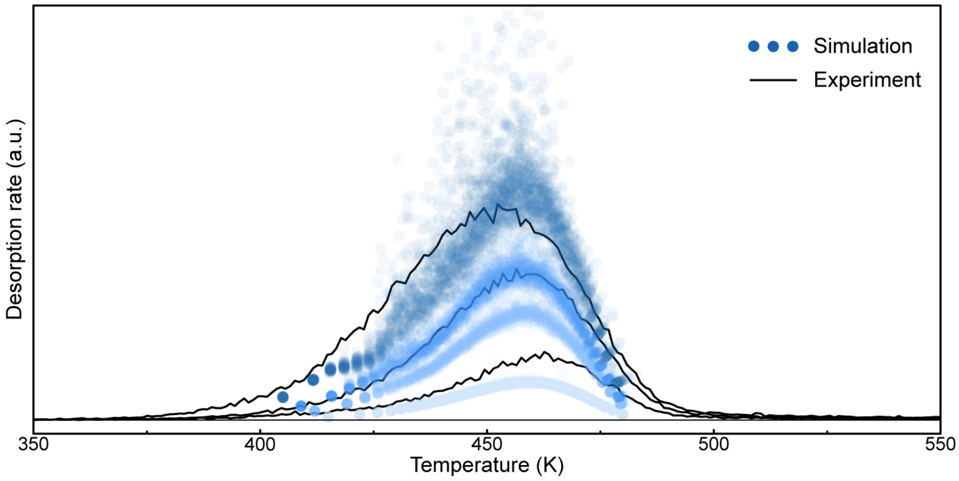

Suppl. Fig. 14 | Simulated and experimental TPD of PEN monolayers adsorbed MoS_2. Solid lines: Experimental TPD traces of PEN (sub-) monolayers adsorbed MoS_2 as published in Ref. 12. Blue dots: Simulated TPD traces of PEN (sub-) monolayers adsorbed on MoS_2 for similar initial coverages to the experimental data. For each simulated coverage, 50 individual experiments were simulated and superimposed transparently in the graph to visualize both clustering and scattering of the Monte Carlo simulations.

To check the validity of our model for intermolecular interactions, we have simulated temperature-programmed desorption (TPD) of PEN adsorbed on MoS_2, since experimental TPD traces are available for reference.[12] For this simulation, we have used the same Buckingham potential parameters for Pauli repulsion and dispersion forces as in the main paper, and we have also determined effective atomic charges for PEN by means of natural bond orbital (NBO) analysis.

In contrast to the desorption simulations conducted in this work for L- and M-F_6PEN, we have allowed molecular diffusion between desorption events and included periodic boundary conditions so that surface coverages can be defined accurately. These changes are computationally highly expensive, which is why the number of molecules per simulated experiment was reduced to only 64. The algorithm for desorption is based on the work of Meng and Weinberg,[13] and is described in detail in Ref. 12 and more briefly in the *Methods* section of the main paper. In comparison to Ref. 12, only the model for the molecules and their interactions was changed, which also required the inclusion of rotational energy of motion, as molecules were previously not treated as extended bodies. As in Ref. 12, an activation energy of desorption of 122 kJ mol^{-1} was chosen together with a prefactor of $5 \cdot 10^{12}$ s^{-1}.

Suppl. Fig. 14 shows simulated TPD traces (blue dots) together with the experimental data (solid lines) from Ref. 12. For the simulation, four different initial coverages of 0.10 ML (lightest blue), 0.30 ML, 0.45 ML, and 0.80 ML (darkest blue) were chosen, which are close to the experimental coverages (0.17 ML, 0.42 ML, and 0.73 ML). For each simulated coverage, 50 individual experiments with 64 molecules each and periodic boundary conditions were conducted. The resulting TPD traces are plotted transparently to show their scattering while also highlighting the clustering of the individual simulated TPD traces through stronger colour due to the overlay.

It is obvious that the simulated TPD traces do not match exactly the experimental data. Nonetheless, the overall trend of a shifting leading edge, as well as the width of the traces, is reproduced rather accurately. This shows that the interaction energies, which cause the shift of the leading edge to lower temperatures with increasing coverage (cf. Ref. 12) are accurately simulated, thus proving the reliability of our model that is also used for L- and M-F_6PEN.

Supplementary references

1. P. E. Hofmann, M. W. Tripp, D. Bischof, Y. Grell, A. L. C. Schiller, T. Breuer, S. I. Ivlev, G. Witte and U. Koert, *Angew. Chem. Int. Ed.*, 2020, **59**, 16501.
2. T. Breuer, M. Klues and G. Witte, *J. Electron Spectrosc. Relat. Phenom.*, 2015, **204**, 102.
3. J. Schwaben, N. Münster, M. Klues, T. Breuer, P. Hofmann, K. Harms, G. Witte and U. Koert, *Chem. Eur. J.*, 2015, **21**, 13758.
4. T. Breuer, T. Maßmeyer, A. Mänz, S. Zoerb, B. Harbrecht and G. Witte, *Phys. Status Solidi RRL*, 2016, **10**, 905.
5. R. Félix, T. Breuer, P. Rotter, F. Widdascheck, B. Eckhardt, G. Witte, K. Volz and K. I. Gries, *Cryst. Growth Des.*, 2016, **16**, 6941.
6. M.-H. Whangbo, J. Ren, S. N. Magonov, H. Bengel, B. A. Parkinson and A. Suna, *Surf. Sci. Lett.*, 1995, **326**, 311.
7. G. Witte, S. Lukas, P. S. Bagus and C. Wöll, *Appl. Phys. Lett.*, 2005, **87**, 263502.
8. M. S. Gordon and M. W. Schmidt in *Theory and applications of computational chemistry: The first forty years*, ed. C. E. Dykstra, Elsevier, Boston, Mass., 2005, p 1167.
9. E. D. Glendening, J. K. Badenhoop, A. E. Reed, J. E. Carpenter, J. A. Bohmann, C. M. Morales, P. Karafiloglou, C. R. Landis and F. Weinhold, *NBO 7.0*, Theoretical Chemistry Institute, University of Wisconsin, Madison, 2018.
10. I. Kröger, B. Stadtmüller, C. Wagner, C. Weiss, R. Temirov, F. S. Tautz and C. Kumpf, *J. Chem. Phys.*, 2011, **135**, 234703.
11. R. A. Scott and H. A. Scheraga, *J. Chem. Phys.*, 1965, **42**, 2209.
12. S. R. Kachel, P. M. Dombrowski, T. Breuer, J. M. Gottfried and G. Witte, *Chem. Sci.*, 2021, **12**, 2575.
13. B. Meng and W. H. Weinberg, *J. Chem. Phys.*, 1994, **100**, 5280.
