# Supplementary material for: Shape control in 2D molecular nanosheets by tuning anisotropic intermolecular interactions and assembly kinetics
Source: Nat Commun. 2023 Mar 21;14:1554. doi: 10.1038/s41467-023-37203-7 (PMC10030871; doi:10.1038/s41467-023-37203-7)
Supplement: Supplementary file 3 — Description of Additional Supplementary Files [file 41467_2023_37203_MOESM3_ESM.pdf]

Description of Additional Supplementary Files

File Name: Supplementary Movie 1
Description: Simulated desorption of an L-F6PEN nanosheet.
